# 1631. Background Incidence Rates of Health Outcomes Relevant to Vaccine Monitoring in Lyme Disease Endemic and Non-endemic US Regions using Administrative Claims Data

**DOI:** 10.1093/ofid/ofad500.1465

**Published:** 2023-11-27

**Authors:** Jill Dreyfus, Swapna Munnangi, Mitchell DeKoven, Camilla Bengtsson, Barbara Correia, Rejane Figueiredo, Sarah Galvin, James H Stark, Michele Zawora, Mark Riddle, Jason Maguire, Qin Jiang, Juan Naredo Turrado, Henrik Svanström, Steven Bailey

**Affiliations:** Pfizer, Inc., New York, New York; IQVIA, Falls Church, Virginia; IQVIA, Falls Church, Virginia; IQVIA, Falls Church, Virginia; IQVIA, Falls Church, Virginia; IQVIA, Falls Church, Virginia; Pfizer, Inc., New York, New York; Pfizer Biopharma Group, Collegeville, Pennsylvania; Pfizer, Inc., New York, New York; Pfizer, Inc., New York, New York; Pfizer Vaccine Clinical Research and Development, Pearl River NY, Pearl River, NY; Pfizer, Collegeville, Pennsylvania; IQVIA, Falls Church, Virginia; IQVIA, Falls Church, Virginia; Pfizer, Inc, New York, New York

## Abstract

**Background:**

A 6-valent vaccine (VLA15) is being tested in clinical trials for the prevention of Lyme disease caused by *Borrelia burgdorferi* sensu lato strains expressing OspA serotypes 1-6. Background incidence rates (IRs) of health outcomes in Lyme disease endemic and non-endemic regions of the US may help to contextualize whether the frequencies of events reported during vaccine clinical trials or post-marketing are consistent with expected population level rates. The objective of this study was to estimate and compare IRs of health outcomes in Lyme disease endemic vs. non-endemic regions using US administrative claims data.

**Methods:**

IQVIA PharMetrics^®^ Plus commercial claims database was used to estimate IRs of 63 outcomes relevant to vaccine safety monitoring in the US during 01/01/2017-12/31/2019. Endemic regions were classified using 3-digit zip codes that overlapped with Lyme disease high incidence counties (10 cases/100,000 persons) according to the CDC. Analyses included all individuals aged ≥ 2 years with ≥ 1 year of enrollment. Outcomes were defined by ICD-10-CM diagnosis codes according to the literature or expert input and required ≥ 1 inpatient or ≥ 2 outpatient claims/codes. Crude IRs and 95% confidence intervals (CIs) were calculated for each outcome and compared between endemic vs. non-endemic regions using IR ratios (IRR).

**Results:**

The study population included 8.7M in endemic and 27.8M in non-endemic regions. Mean age was slightly higher in endemic (37.7 yrs [SD=18.9]) vs. non-endemic (36.8 yrs [SD=19.5]) cohorts, and 51% in both cohorts were female. **Table 1** provides a summary of the IRs and IRRs for the 10 highest and 10 lowest ranking IRRs for health conditions by endemic region status. IRRs (95% CI) ranged from a low of 0.74 (0.71, 0.78) for systemic lupus erythematosus to a high of 2.14 (1.93, 2.37) for meningoencephalitis.

**Figure:**
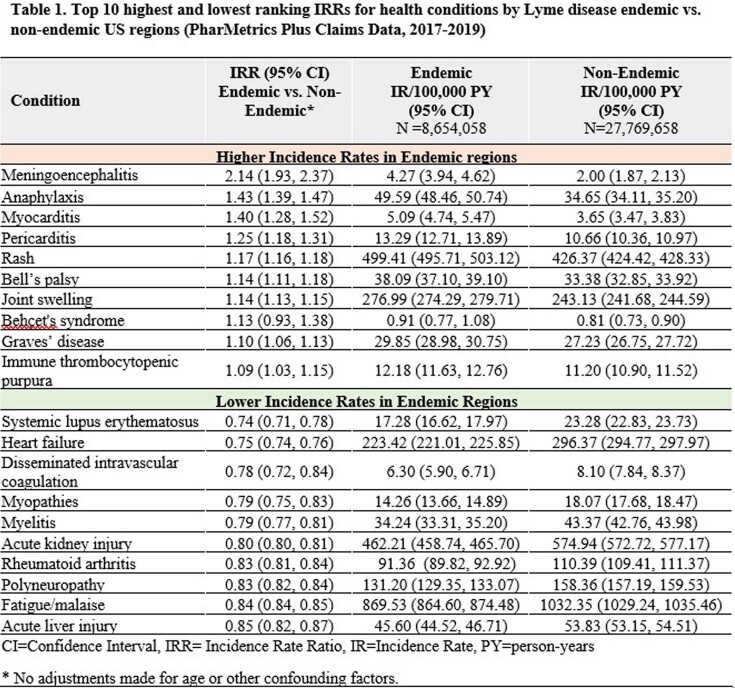

**Conclusion:**

This study identified potential differences between Lyme endemic and non-endemic regions of the US in background IRs of health conditions in vaccine safety monitoring. Differences in background IRs between endemic and non-endemic regions should be considered when contextualizing possible safety signals in clinical trials and post-marketing.

**Disclosures:**

**Jill Dreyfus, PhD, MPH**, Pfizer, Inc.: Employment|Pfizer, Inc.: Stocks/Bonds **Swapna Munnangi, PhD**, IQVIA: Employment **Camilla Bengtsson, PhD**, IQVIA: Employment|Pfizer, Inc: Advisor/Consultant **Barbara Correia, PhD**, IQVIA: Employee|Pfizer, Inc.: Advisor/Consultant **Rejane Figueiredo, PhD**, IQVIA: Biostatistician **Sarah Galvin, BS**, Pfizer, Inc.: Employment **James H. Stark, PhD**, Pfizer: Employee|Pfizer: Stocks/Bonds **Michele Zawora, MD, FAAFP**, Pfizer: Employment|Pfizer: Stocks/Bonds **Mark Riddle, MD, DrPH**, Pfizer: Employee salary **Jason Maguire, MD**, Pfizer, Inc.: Employee|Pfizer, Inc.: Stocks/Bonds **Qin Jiang, PhD**, Pfizer: Employee|Pfizer: Employee|Pfizer: Stocks/Bonds|Pfizer: Stocks/Bonds **Juan Naredo Turrado, MS**, IQVIA: Employee **Steven Bailey, MD, MPH, MBA**, Pfizer, Inc.: Employment

